# Conditional Protein Splicing Switch in Hyperthermophiles through an Intein-Extein Partnership

**DOI:** 10.1128/mBio.02304-17

**Published:** 2018-01-30

**Authors:** Christopher W. Lennon, Matthew Stanger, Nilesh K. Banavali, Marlene Belfort

**Affiliations:** a Department of Biological Sciences, University at Albany, Albany, New York, USA; b RNA Institute, University at Albany, Albany, New York, USA; c Department of Biomedical Sciences, School of Public Health, University at Albany, Albany, New York, USA; d Division of Genetics, Laboratory of Computational and Structural Biology, Wadsworth Center, NYS Department of Health, Albany, New York, USA; UCLA School of Medicine

**Keywords:** Posttranslational regulation, recombinase, hyperthermophile, mobile genetic elements

## Abstract

Inteins are intervening proteins that undergo an autocatalytic splicing reaction that ligates flanking host protein sequences termed exteins. Some intein-containing proteins have evolved to couple splicing to environmental signals; this represents a new form of posttranslational regulation. Of particular interest is RadA from the archaeon *Pyrococcus horikoshii*, for which long-range intein-extein interactions block splicing, requiring temperature and single-stranded DNA (ssDNA) substrate to splice rapidly and accurately. Here, we report that splicing of the intein-containing RadA from another archaeon, *Thermococcus sibericus*, is activated by significantly lower temperatures than is *P. horikoshii* RadA, consistent with differences in their growth environments. Investigation into variations between *T. sibericus* and *P. horikoshii* RadA inteins led to the discovery that a nonconserved region (NCR) of the intein, a flexible loop where a homing endonuclease previously resided, is critical to splicing. Deletion of the NCR leads to a substantial loss in the rate and accuracy of *P. horikoshii* RadA splicing only within native exteins. The influence of the NCR deletion can be largely overcome by ssDNA, demonstrating that the splicing-competent conformation can be achieved. We present a model whereby the NCR is a flexible hinge which acts as a switch by controlling distant intein-extein interactions that inhibit active site assembly. These results speak to the repurposing of the vestigial endonuclease loop to control an intein-extein partnership, which ultimately allows exquisite adaptation of protein splicing upon changes in the environment.

## IMPORTANCE

Inteins are mobile genetic elements that interrupt coding sequences (exteins) and are removed by protein splicing. They are abundant elements in microbes, and recent work has demonstrated that protein splicing can be controlled by environmental cues, including the substrate of the intein-containing protein. Here, we describe an intein-extein collaboration that controls temperature-induced splicing of RadA from two archaea and how variation in this intein-extein partnership results in fine-tuning of splicing to closely match the environment. Specifically, we found that a small sequence difference between the two inteins, a flexible loop that likely once housed a homing endonuclease used for intein mobility, acts as a switch to control intein-extein interactions that block splicing. Our results argue strongly that some inteins have evolved away from a purely parasitic lifestyle to control the activity of host proteins, representing a new form of posttranslational regulation that is potentially widespread in the microbial world.

## INTRODUCTION

**FIG 1:**
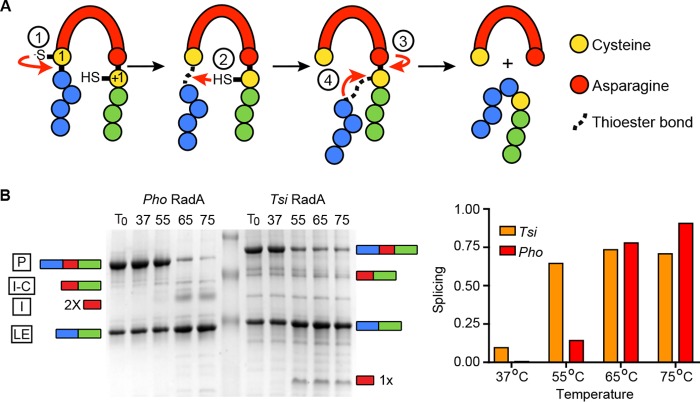
Splicing mechanism and temperature requirements of *P. horikoshii* and *T. sibericus* RadA proteins for splicing in native exteins. (A) Schematic of class I protein splicing mechanism, where both catalytic nucleophiles are cysteines. Details of each reaction step are described in the text. (B) *P. horikoshii* and *T. sibericus* RadA splicing requires an elevated temperature in native exteins. (Left) Representative data are shown for samples incubated for 30 min at the indicated temperatures. Samples were separated by SDS-PAGE and stained with Coomassie, and levels of each species were determined using densitometry. A cartoon representation of on-pathway intein splicing with native exteins to the side shows precursor (P), ligated exteins (LE), and intein (I) and off-pathway intein–C-extein product (I-C). The *P. horikoshii* RadA intein runs as a dimer (13), whereas the *T. sibericus* RadA intein resolves as a monomer. 2× indicates an intein dimer, and 1× indicates an intein monomer. (Right) Bar graph showing quantification of the samples shown in the schematic on the left.

Inteins, or ***int***ervening prot***eins***, are mobile genetic elements that are translated within host proteins and removed through protein splicing (1–3). In this process, the intein autocatalytically removes itself by breaking two peptide bonds and joining the flanking sequences (called N- and C-exteins) via a peptide bond, leaving the host protein (ligated exteins) without any trace of where the intein once resided (Fig. 1A).

Inteins are widespread in nature, and they are found in all three domains of life as well as in phages and viruses (4, 5). Inteins are particularly abundant in bacteria and archaea, as they are present in nearly one-quarter and one-half of reference sequences, respectively (4). Not only are inteins widespread, but also they often are found within proteins important or essential to organism fitness. Inteins cluster to particular functional classes of proteins, with over 70% found in ATP-binding proteins and almost two-thirds found in proteins involved in DNA replication, recombination, and repair (RRR). Remarkably, inteins have even been independently acquired by evolutionarily distinct bacterial and archaeal proteins with equivalent functions (4). Inteins are generally found in sites critical to host protein function, such as in active sites or protein-protein interaction interfaces.

Inteins have long been considered parasitic elements that provide no benefit to the host organism (6). Recent work has refuted this notion by demonstrating that the splicing of several inteins can be regulated by a variety of environmental cues relevant to the survival of the host organisms or crucial to host protein function (7–13). These conditions include pH, redox, reactive oxygen-nitrogen species, salt, temperature, and even host protein substrate. This environmental control suggests that in some cases, inteins have evolved to benefit the host through posttranslational regulation of protein function. Furthermore, bioengineers can develop inteins that will splice in response to a variety of stimuli to control ligation or cleavage reactions (14–16), begging the question of how nature may have employed similar tactics as a mechanism of posttranslational control in intein-containing proteins.

Our group has discovered a landmark example of conditional protein splicing in the RadA recombinase from the deep sea hyperthermophilic archaeon *Pyrococcus horikoshii*, a member of the highly conserved RecA/Rad51 homologous recombinase family (17). What makes this example striking is that splicing in native exteins requires high temperatures (~75°C) to proceed, while splicing in foreign exteins occurs rapidly at low temperature (~25°C) (9). Based on structure modeling and mutagenesis, we determined that splicing inhibition was due to intein-C-extein interactions between residues well-separated in sequence that must be broken for splicing to proceed (9). Our findings on *P. horikoshii* RadA became even more remarkable when we discovered that splicing can be activated by the substrate of the host protein, single-stranded DNA (ssDNA). In the first example of such regulation, ssDNA, the cell’s signal that RadA recombinase activity is needed, dramatically accelerates and improves the accuracy of *P. horikoshii* RadA splicing at all temperatures tested (13).

Nature has provided an interesting ortholog of *P. horikoshii* RadA in *Thermococcus sibiricus*. *T. sibericus* is an archaeon that is similar in lifestyle to *P. horikoshii* but, whereas *P. horikoshii* grows between 70 and 102°C (18), *T. sibericus* survives at significantly lower temperatures, between 40 and 88°C (19). While the difference in growth temperatures between these two archaea is substantial, *P. horikoshii* and *T. sibericus* RadA proteins are highly similar at the amino acid level, including an identical intein insertion site within the P-loop of the RadA ATPase domain. Both *P. horikoshii* and *T. sibericus* RadA naturally possess mini-inteins that lack the homing endonuclease domain, which is often present and presumed to be required for spread into non-intein-containing alleles through homing (4, 5, 20).

Here, we compare the splicing of intein-containing RadA proteins from *T. sibericus* and *P. horikoshii*. We found that, whereas splicing is accelerated at a high temperature only in the native exteins for both RadA inteins, the temperature required is different and corresponds closely to those where the organisms are found in nature. Through analysis of a region poorly conserved between the *T. sibericus* and *P. horikoshii* inteins, we describe the first substitution that disrupts splicing in native, but not foreign, extein contexts, and this defect can be rescued by substrate ssDNA. We propose that this region, a flexible loop that previously housed a homing endonuclease and that is located between conserved splicing domains, behaves as a hinge that helps disrupt an inhibitory intein-extein partnership, effectively acting as a switch to turn on splicing.

## RESULTS

### *P. horikoshii* and *T. sibericus* RadA splicing is differentially regulated by temperature.

The canonical class I intein splicing mechanism proceeds in four steps (Fig. 1A). In the first step, the first residue of the intein (known as the 1 position; either a cysteine or serine) performs a nucleophilic attack on the preceding peptide bond, forming a (thio)ester linkage. Next, the first residue of the C-extein (known as the +1 position; either a cysteine, serine, or threonine) performs a nucleophilic attack on the (thio)ester resulting from step 1 and forms a branched intermediate. Third, an asparagine found at the end of the intein cyclizes to liberate the intein. Finally, the (thio)ester linking the N- and C-exteins rearranges to form a peptide bond. Off-pathway cleavage reactions can occur as well, in which either the N- or C-extein is released prior to extein ligation, resulting in products of no known utility to the cell.

Previously we found that when we removed the first 115 residues corresponding to the N-terminal domain (NTD) of *P. horikoshii* RadA, leaving the ATPase and intein intact, splicing acceleration by temperature and ssDNA was unaffected (13). We therefore used ΔNTD constructs of *P. horikoshii* and *T. sibericus* RadA that were expressed and purified from *Escherichia coli* whenever splicing was examined in the native extein context; we refer to these constructs as wild type. First, we examined whether *T. sibericus* RadA intein splicing was stimulated by increasing temperature in the context of native exteins, as is the case for *P. horikoshii* RadA (9), as this would indicate conservation of this mode of regulation in nature. Following overexpression and His tag purification of *T. sibericus* and *P. horikoshii* RadA under identical conditions, a significant amount of unspliced RadA precursor was isolated in both cases. We next incubated purified *T. sibericus* and *P. horikoshii* RadA proteins at temperatures ranging from 37°C to 75°C and measured splicing after 30 min. Whereas *T. sibericus* RadA splicing was also activated by increasing temperatures, the initiation of splicing occurred at a lower temperature than for *P. horikoshii* RadA (Fig. 1B). (Throughout figures *P. horikoshii* is referred to as Pho and *T. sibericus* is referred to as Tsi.)

**FIG 2:**
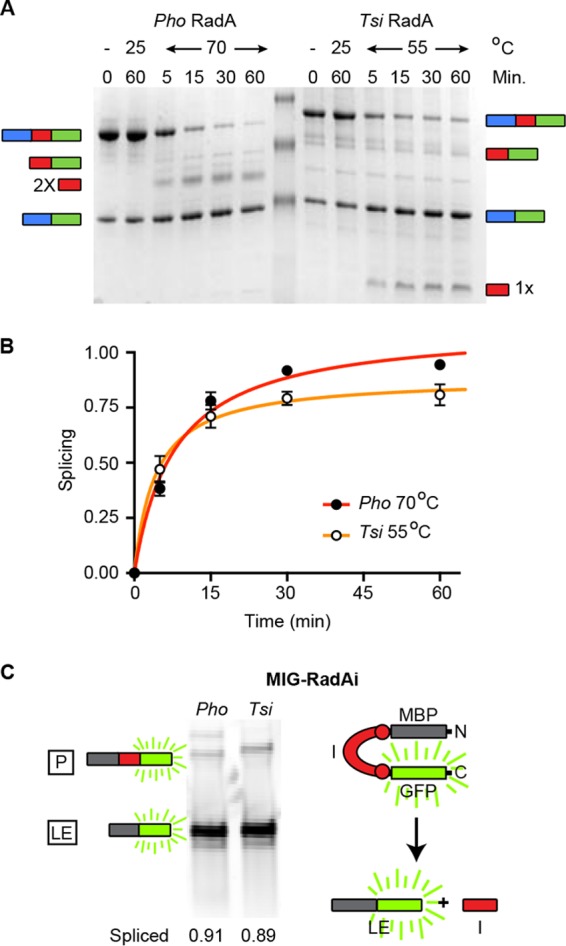
*P. horikoshii* and *T. sibericus* RadA splicing at different temperatures. (A) Splicing time course of *T. sibericus* RadA at 55°C and *P. horikoshii* RadA at 70°C at the indicated times. Additionally, each protein was incubated at 25°C for 1 h. Samples in panels A and B were separated, analyzed, and identified as described for Fig. 1. (B) Scatter plot of splicing results shown in panel A. Points are averages of three independent experiments, with error bars showing standard deviations. Error bars smaller than the size of the point are not shown. A small portion of the *T. sibericus* RadA precursor does not splice, even after prolonged incubation at an elevated temperature, which is likely a result of a catalytically inactive alternative conformation following purification. The initial rates of splicing are indicated in the text. (C) *P. horikoshii* and *T. sibericus* RadA splicing in the foreign MIG extein context. Samples were incubated at 25°C for 1 h following expression. Included is a cartoon representation of the MIG reporter. Samples were separated by SDS-PAGE, and in-gel GFP fluorescence was measured.

To establish the precise difference in temperature of splicing between *T. sibericus* and *P. horikoshii* RadA proteins, we determined the temperatures at which the rates of splicing were approximately the same for the two proteins, which was 70°C and 55°C for *P. horikoshii* RadA and *T. sibericus* RadA, respectively. We thus incubated *P. horikoshii* RadA at 70°C and *T. sibericus* RadA at 55°C and measured splicing at various times over 1 h (Fig. 2A). We also incubated each protein at 25°C for the duration of the time course as a control to ensure that temperature was responsible for activation of splicing. The initial rates of splicing were approximately equal under these conditions (7.7 × 10^−2^/min for *P. horikoshii* and 9.4 × 10^−2^/min for *T. sibericus*) (Fig. 2B), demonstrating at least a 15°C shift in splicing activation temperature. This 15°C difference agrees closely with the optimal (20°C) and maximal (14°C) growth temperature difference between *T. sibericus* and *P. horikoshii* (18, 19).

### *T. sibericus* RadA does not require elevated temperature for splicing in foreign exteins.

We next asked if increased temperature was needed to stimulate *T. sibericus* RadA splicing because of something inherent to the intein itself, or whether this was dependent on its being in the native extein context, as is the case for *P. horikoshii* RadA (9). We used our foreign extein splicing reporter, MIG (*M*BP-*i*ntein-*G*FP), which contains the maltose binding protein (MBP) followed by the intein (flanked on each side by 10 native extein residues), followed by green fluorescent protein (GFP). After expression in *E. coli*, *T. sibericus* and *P. horikoshii* RadA inteins in the MIG reporter were reincubated for 1 h at 25°C, and the precursor and products were measured based on in-gel fluorescence of C-extein GFP. Both inteins spliced rapidly and accurately without incubation at higher temperatures (Fig. 2C), indicating that splicing can proceed at low temperatures but that processing is blocked in the native extein context.

### Tuning of thermoregulated splicing by intein–C-extein interactions.

**FIG 3:**
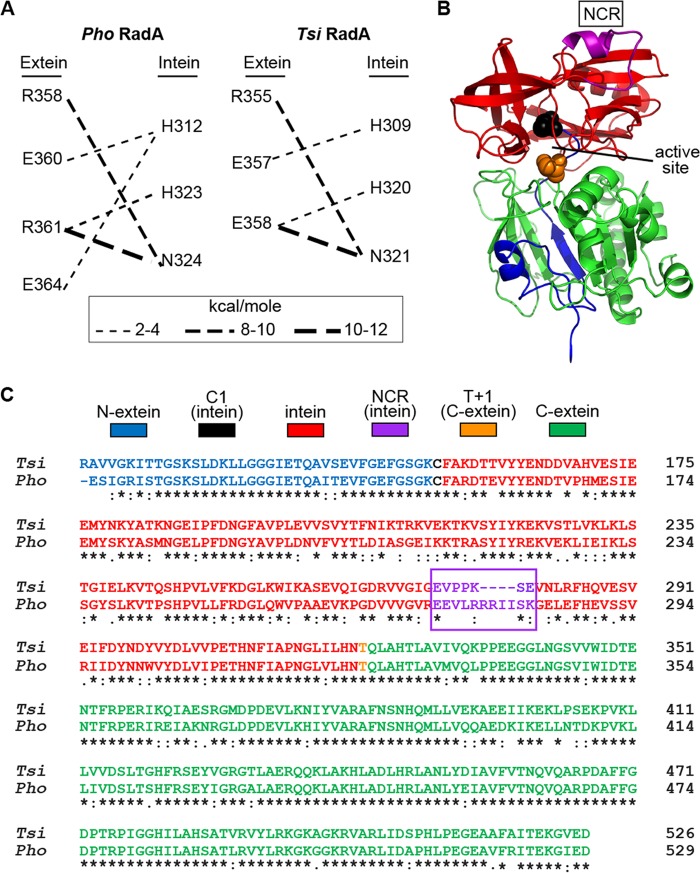
Predictions for intein-extein interactions, model of the *P. horikoshii* RadA precursor, and alignment of the *P. horikoshii* and *T. sibericus* RadA proteins. (A) Predicted intein-extein side chain interactions greater than 1 kcal/mol. A E364-H312 side chain interaction is predicted in the *P. horikoshii* RadA precursor models, but not between the corresponding Q361-H309 of *T. sibericus* RadA. (B) Molecular model of the *P. horikoshii* RadA precursor residues 116 to 529. Pymol was used to generate the image from a previously rendered model (9). The sequence and model are colored according to the color key of panel C. (C) Residues 116 to 529 of *P. horikoshii* RadA and residues 116 to 526 of *T. sibericus* RadA were aligned using Clustal Omega. An asterisk indicates absolute conservation, a colon indicates strong conservation, a period indicates weak conservation, and an empty space indicates no conservation.

We posit that the difference between the temperature required to initiate splicing of *P. horikoshii* and that for *T. sibericus* RadA in native exteins (Fig. 1B and 2A) results from variable intein–C-extein interactions. We previously assessed these interactions by modeling the *P. horikoshii* RadA precursor protein, based on available structures, followed by extensive confirmatory mutagenesis (9). Several residues in the C-extein at the predicted intein interface were replaced with alanine, resulting in disruption of thermoregulated splicing (9). One of these residues of the *P. horikoshii* RadA C-extein, E364, was predicted to interact with the H312 side-chain of the intein (Fig. 3A) (9). Interestingly, the residue corresponding to *P. horikoshii* RadA E364 in *T. sibericus* RadA is Q361, which is similar in size but lacks a side-chain negative charge. Following modeling of the *T. sibericus* RadA precursor, there was no predicted side chain interaction between corresponding *T. sibericus* RadA residues (H309 and Q361 average, −0.1 kcal/mol), compared to similarly minimized *P. horikoshii* RadA precursor models (H312 and E364 average, −2.7 kcal/mol; see Materials and Methods for construction of RadA precursor models). When examining the intein-extein side-chain interactions with energies greater than −1 kcal/mol, only this predicted interaction was variable between *T. sibericus* and *P. horikoshii* RadA (Fig. 3A). We previously replaced *P. horikoshii* RadA E364 with alanine, measured splicing in response to temperature, and observed that this mutation allowed splicing to proceed ~5-fold faster than with the wild type at 55°C (9).

### *P. horikoshii* and *T. sibericus* RadA are divergent in the endonuclease loop of the intein.

While the E364A substitution led to a substantial increase in *P. horikoshii* RadA splicing, it was slower than *T. sibericus* RadA at 55°C (9), indicating that C-extein variation between *P. horikoshii* and *T. sibericus* RadA alone may not fully explain the observed difference in thermoregulated splicing. We hypothesize that something inherent to the intein must also play a role in temperature-induced splicing, possibly by controlling intein-extein interactions. We therefore focused on sequence differences to better understand the observed variation in thermoregulated splicing. One region within the inteins of *P. horikoshii* and *T. sibericus* RadA stands out as highly variable, both in sequence and length, and we call this the nonconserved region (residues 273 to 283 of *P. horikoshii* RadA and residues 274 to 280 of *T. sibericus* RadA). Both *P. horikoshii* and *T. sibericus* RadA house mini-inteins, and the NCR is located between conserved splicing blocks, where the homing endonuclease is located in full-length inteins. Using our previously generated model of the *P. horikoshii* RadA precursor (9), we found that the NCR is distant from both catalytic intein splicing residues and the intein–C-extein interaction interface (Fig. 3B). While there is considerable sequence divergence in the NCR, *T. sibericus* and *P. horikoshii* RadA as a whole are highly similar. Comparison of the amino acid sequences of the *T. sibericus* and *P. horikoshii* RadA precursors, including ATPase domains and inteins, shows that they are 75.8% identical and 92.7% similar based on Clustal Omega analysis (21) (Fig. 3C). Not surprisingly, given the high level of conservation between the recombinases RadA, eukaryotic Rad51, and bacterial RecA (22), the extein sequence is more conserved than is the intein. Extein residues are 85.5% identical and 97.1% similar, whereas the intein residues are 69.8% identical and 84.3% similar.

### The *P. horikoshii* intein NCR is dispensable for splicing in foreign exteins but critical in native exteins.

The structure of the *P. horikoshii* RadA intein was previously solved both by both X-ray crystallography and nuclear magnetic resonance in the absence of native exteins (23). Interestingly, residues 272 to 285 of *P. horikoshii* RadA were disordered (residues 120 to 133 of the intein), prompting those authors to delete intein residues 273 to 282, mutate residue 283 from lysine to asparagine, and resolve the crystal structure of this intein. The structure of the minimized *P. horikoshii* RadA intein was very similar to that of the full-length intein, but it lacked the disordered loop and allowed the intein active site to be visualized at improved resolution (23). Splicing of the minimized intein was indistinguishable from the wild type in a foreign extein context (different from our MIG splicing reporter), prompting those authors to conclude that this region was dispensable for splicing and simply the remnant of the once-present homing endonuclease (23).

**FIG 4:**
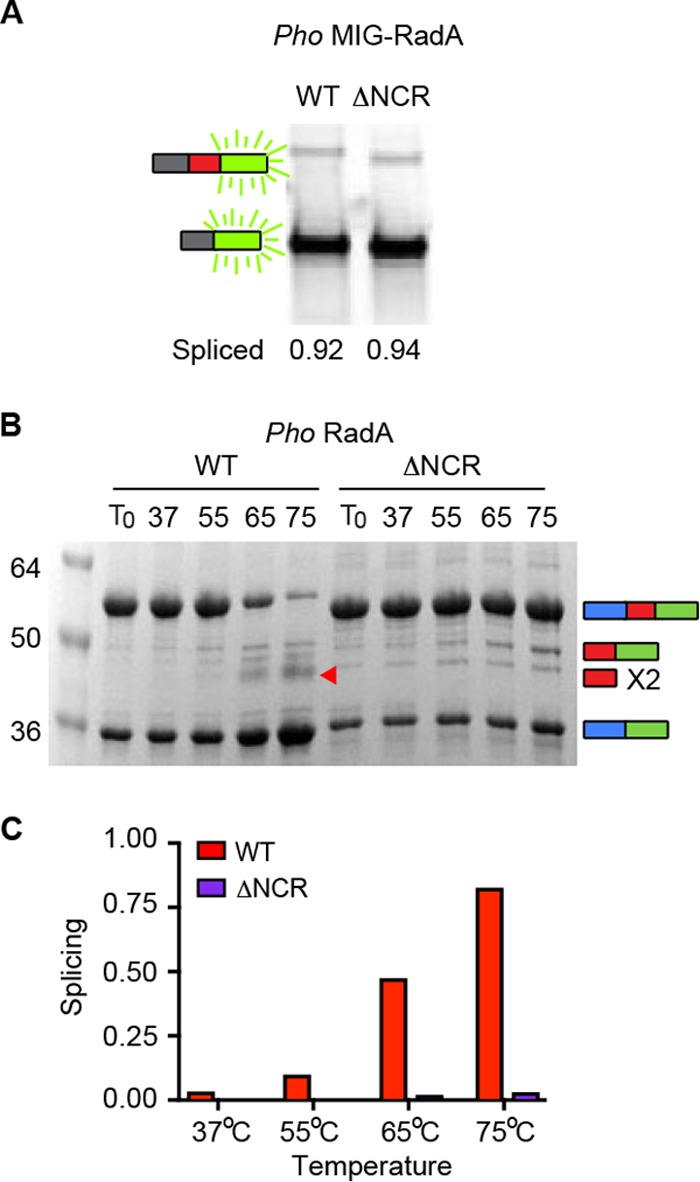
The *P. horikoshii* RadA NCR is only critical to splicing in native exteins. (A) *P. horikoshii* wild-type and ΔNCR RadA protein splicing in the foreign MIG extein context are indistinguishable. Samples were incubated for 1 h at 25°C following expression. (B) *P. horikoshii* RadA ΔNCR splicing is defective in the native extein context. Samples were incubated for 30 min at the temperatures indicated. Samples in panels A and B are representative examples and were separated, analyzed, and identified as described for Fig. 1. The red arrow indicates free intein, observed only in WT splicing. (C) Bar graph illustrating quantification of samples of panel B.

To determine if the NCR influenced splicing in our foreign extein MIG reporter, we deleted residues 273 to 283 of the *P. horikoshii* RadA within the intein (ΔNCR, residues 121 to 131 of the intein) and compared its splicing to the wild-type *P. horikoshii* RadA intein based on in-gel fluorescence from the C-extein–GFP (9). After expression and incubation at 25°C for 1 h, both wild-type and the ΔNCR protein were >90% spliced in MIG (Fig. 4A). This result fit perfectly with previous findings on splicing in the foreign extein context (23).

To investigate any possible role of the NCR in the native extein context, we deleted this region of the *P. horikoshii* RadA intein within the native exteins and incubated the *P. horikoshii* RadA wild type and *P. horikoshii* RadA ΔNCR protein at temperatures ranging from 37°C to 75°C for 30 min. Strikingly, splicing of the *P. horikoshii* RadA ΔNCR protein was strongly inhibited at all temperatures tested, even at 75°C, at which nearly all *P. horikoshii* RadA wild-type was spliced after 30 min (Fig. 4B and C). Even over a 3-h time course at 75°C, little accurate splicing was observed for the *P. horikoshii* RadA ΔNCR protein; rather, off-pathway N-terminal cleavage and disulfide bond formation between precursors at C1 were observed (see Fig. S1 and S2 in the supplemental material). Together, these results demonstrated the first example of an intein mutant that was conditionally defective for splicing in native but not foreign exteins.

### Splicing of the *P. horikoshii* RadA intein NCR deletion is rescued by ssDNA.

**FIG 5:**
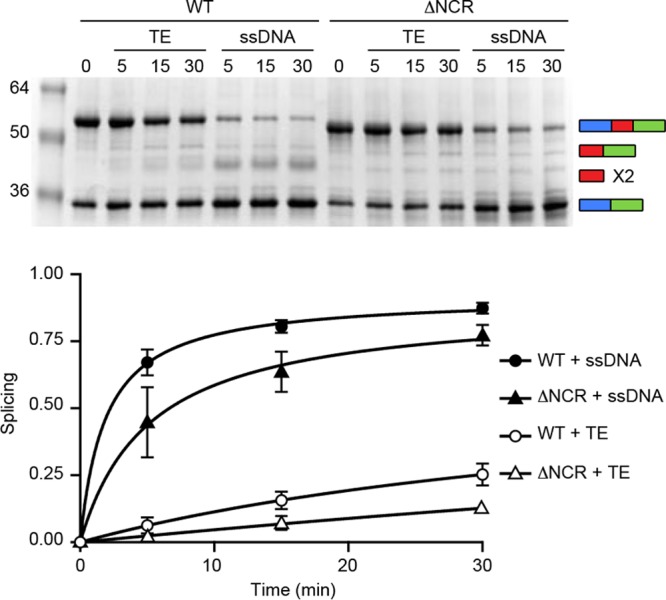
The *P. horikoshii* RadA ΔNCR splicing defect can be rescued by an ssDNA substrate. Samples were incubated at 63°C for the indicated times. (Top) Gel; (bottom) corresponding scatter plot. While splicing is largely on-pathway for the *P. horikoshii* RadA ΔNCR in the presence of ssDNA, as indicated by the increase in ligated exteins, we did not observe free intein dimer, which we attribute to poor resolution on the SDS-PAGE. Each point is the average of three independent experiments, with standard deviations shown by the error bars. Error bars smaller than the size of the point are not shown. Samples were separated, analyzed, and identified as described for Fig. 1.

As described above, a natural RadA substrate, ssDNA, accelerates wild-type *P. horikoshii* RadA splicing by nearly 50-fold, as well as dramatically improving the accuracy of splicing (13). We therefore asked if ssDNA could alleviate the splicing defect caused by the NCR deletion in *P. horikoshii* RadA. At 63°C, both wild-type and the ΔNCR *P. horikoshii* RadA spliced little over a 30-min time course in the absence of ssDNA, especially in the case of the ΔNCR *P. horikoshii* RadA protein, for which virtually no new ligated exteins were generated. Strikingly, the splicing rate and accuracy were largely restored by addition of substrate ssDNA (Fig. 5), demonstrating that the ΔNCR *P. horikoshii* RadA can achieve a catalytically competent conformation. Furthermore, ssDNA binding is sufficient to overcome NCR control of thermoregulated splicing in the native extein context, indicating that this mutant is specifically defective for temperature-activated splicing.

## DISCUSSION

Splicing regulation of intein-containing proteins provides an attractive posttranslational mechanism to inhibit protein activity until conditions are ideal for function. Here, we have shown that while both *P. horikoshii* and *T*. *sibericus* RadAs require an elevated temperature to splice in the native extein context, the temperature needed corresponds closely to ambient temperatures in the natural environments of *T. sibericus* and *P. horikoshii*. We propose that the tailoring of splicing to a particular temperature is regulated by a variable intein-extein partnership. This natural example of conditional protein splicing, where formation of a functional host protein is exquisitely tuned to the evolutionary niche of the organism, demonstrates the power of inteins to impart regulation on protein activity to the potential benefit of the host organism. However, it is possible that conditional protein splicing evolved as a mechanism to minimize damage from nonspecific and off-pathway splicing. In either case, conditional protein splicing could be beneficial to the host.

Given the position of the NCR remote from the active site in what is a vestigial endonuclease loop in the structure of the intein (Fig. 3B), it is likely that this region influences the activity of distant residues. Structural work has shown that the *P. horikoshii* RadA intein NCR is highly flexible (23), leading us to posit that the NCR functions to allow flexibility between conserved splicing domains, much like a hinge, to promote splicing. In support of this role for the NCR, computational studies with the *Mycobacterium tuberculosis* RecA intein found that the flexibility in the endonuclease loop region is higher in the active vs. inactive intein and that linker length influences dynamics (24).

**FIG 6:**
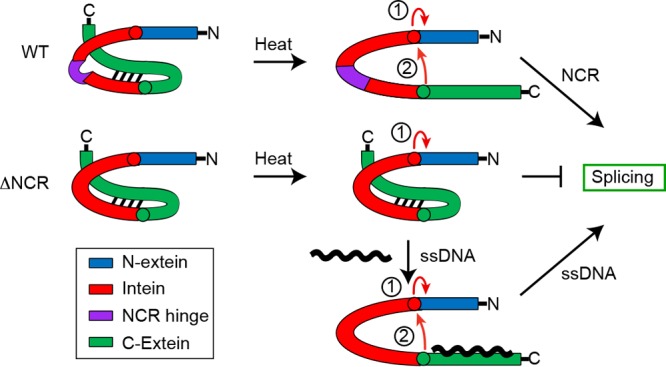
Model describing a role for the intein NCR in control of intein-extein interactions. Color coding of intein, N-extein, and C-extein is indicated in the key. Interactions between the intein and C-extein block splicing of the wild-type and ΔNCR RadA at low temperatures are shown. High temperatures can break these interactions in an NCR-dependent manner, whereby the NCR acts as a flexible hinge to switch on splicing. In contrast, ΔNCR maintains inhibitory intein–C-extein interactions. ssDNA, which accelerates and improves the accuracy of *P. horikoshii* RadA splicing, compensates for the absence of the NCR, interacting with the C-extein to break interactions with the intein, and promoting the catalytically competent state. The first two steps of the splicing reaction are indicated by red arrows (described in the text and the legend for Fig. 1A). We propose that step 2 of the reaction is blocked in the ΔNCR RadA protein in the absence of ssDNA.

To explain why the *P. horikoshii* RadA NCR is needed for splicing in the native extein context but not in two foreign extein reporters (Fig. 4A) (23), we considered what is unique to splicing in native exteins, namely, an interaction between the intein and C-extein that must be broken to allow for splicing to proceed (9). In the absence of the NCR, we propose that this interaction is stable even at an increased temperature due to the lack of intein flexibility conferred by the NCR, blocking the availability of other intein residues for catalysis (Fig. 6) (9). Specifically, key residues at the C-terminal splice site would be unavailable due to proximal interactions with the C-extein (Fig. 3A and 6) (9). This model is supported by our observation that the ΔNCR *P. horikoshii* RadA C-1 is still highly reactive, resulting in increased off-pathway splicing compared to the wild type, namely, N-terminal cleavage (Fig. 4B; Fig. S1) and disulfide bond formation at C-1 between adjacent RadA precursors (Fig. S2). In the presence of ssDNA, C-extein binding to the substrate overrides the intein–C-extein interactions and releases the intein to achieve a catalytically competent state (Fig. 6). The slow, off-pathway splicing is also alleviated (Fig. 5; Fig. S1 and S2). Interestingly, we saw some evidence of *P. horikoshii* RadA ΔNCR splicing to form ligated exteins following expression in *E. coli* (Fig. 4 and 5; Fig. S1 and S2), in contrast to our observations *in vitro* in the absence of ssDNA, suggesting the presence of elements in the cellular environment, possibly the low levels of ssDNA when there is fast growth, that can promote splicing.

10.1128/mBio.02304-17.2 FIG S1  *P. horikoshii* RadA ΔNCR forms primarily off-pathway products. Reactions were performed at 75°C for the indicated times. (Top) Stack plot showing the level of splicing precursor (PC) and products (PC-PC, precursor dimer; I-C, intein–C-extein; Ix2, intein dimer; LE, ligated exteins), taken as 100% over time. (Bottom) Gel for the data shown in the stack plot. Samples were separated, analyzed, and identified as described for Fig. 1. Download FIG S1, TIF file, 0.7 MB. Copyright © 2018 Lennon et al. 2018 Lennon et al. This content is distributed under the terms of the Creative Commons Attribution 4.0 International license.

10.1128/mBio.02304-17.3 FIG S2  The *P. horikoshii* RadA ΔNCR forms an intermolecular precursor-precursor disulfide bond at intein C-1 residues. Following incubation of the *P. horikoshii* RadA ΔNCR for 25 min at 75°C to generate the high-molecular-weight product, the reaction mixture was divided, and either DTT, BME, TCEP, HA, or water was added. To allow for disulfide reduction, reactions were run at 25°C for 80 min. Reaction mixtures were separated on 8-to-16% SDS-PAGE gels and stained with Coomassie. Download FIG S2, TIF file, 0.2 MB. Copyright © 2018 Lennon et al. 2018 Lennon et al. This content is distributed under the terms of the Creative Commons Attribution 4.0 International license.

We thus demonstrated that the NCR of the *P. horikoshii* RadA intein is crucial to the rate and accuracy of splicing in native exteins but is dispensable in the foreign extein context. To our knowledge, this is the first such intein-extein connection described. This finding is of importance beyond the implications for RadA splicing regulation and demonstrates the close relationship between inteins and their native exteins, supporting a complex coevolutionary scenario which can serve to control splicing in response to environmental signals (25, 26).

This is not the first work to demonstrate that intein substitutions in the endonuclease loop can have a profound effect on splicing despite the distance from the active site. In a selection effort to generate hyperactive variants of the RecA intein from *M. tuberculosis*, our group previously found that the most common site of mutation was in this loop, and we hypothesized that substitutions led to a “ripple effect” on the environment of the catalytic intein residues (27). The endonuclease loop has also been used as a site for insertion of foreign proteins that can be engineered to splice in response to conformational changes triggered by small-molecule binding (15, 16). Further support was recently garnered by Iwai and colleagues, who demonstrated via a combination of structural and biochemical techniques the importance of endonuclease loop dynamics for the splicing of the TFIIB inteins from *Methanococcus jannaschii* and *Methanocaldococcus vulcanius* M7 (28).

Beyond the mechanistic implications of *P. horikoshii* RadA ΔNCR splicing rescue by ssDNA, these findings could be useful in biotechnology. Virtually no on-pathway splicing of the *P. horikoshii* RadA ΔNCR occurred in the absence of substrate ssDNA *in vitro* at all temperatures tested (Fig. 4B and 5; Fig. S2), but splicing is rapid and accurate when incubated in the presence of ssDNA (Fig. 5). This system could be engineered into an on/off splicing switch or sensor to generate ligated exteins tightly controlled by the addition of ssDNA or DNA damage.

Because the NCR is outside the highly conserved blocks of the intein required for splicing, there exists greater plasticity for adaptation within this region for fine-tuning of splicing in a specific extein context while maintaining conservation of the splicing blocks critical to catalysis. Thus, an intein region that was considered to be irrelevant due to lack of conservation, that was located in the truncated endonuclease region not required for splicing, has been repurposed via evolution to act as a switch to promote splicing in the native extein context. Our results as a whole demonstrate the contribution of both intein-extein interactions and a nonconserved intein region to conditionally control the progression of splicing in response to environmental cues in native exteins. These findings speak strongly to the complex evolutionary relationship between these mobile elements and the host organisms they invade, and our findings also demonstrate the potential for nature to utilize protein splicing as a means of posttranslational regulation.

## MATERIALS AND METHODS

### Plasmid construction.

*T. sibericus* RadA (Δ1-115 codons) was PCR amplified using primers IDT4699 (5′-GGTGGTGGATCCGAGGGCGGTTGTTGGGAAG-3′) and IDT4670 (5′-GGTGGTCTCGAGTCAATCTTCGACACCTTTCTCCG-3′) from *T. sibericus* genomic DNA (DSM 12597; Leibniz-Institut DSMZ) and cloned into pET45b by using BamHI/XhoI restriction sites in-frame with an N-terminal His tag. Plasmid pACYC-*T. sibericus* MIG-RadAi was constructed by amplifying the intein sequence plus 10 flanking codons on each side (codons 144 to 331 of full-length *T. sibericus* RadA) and then cloned into SphI/ClaI sites from the previously constructed pACYC-*M. tuberculosis* MIG-SufBi (10). The *P. horikoshii* NCR (codons 273 to 283 of full-length *P. horikoshii* RadA) was deleted from pET45b-*P. horikoshii* RadA and *P. horikoshii* MIG-RadAi by using Q5 mutagenesis (New England Biolabs) with primers IDT5040 (5′-CCTAACACCAACAACAAC-3′) and IDT5041 (5′-GAAGAACTTGAATTCCATG-3′). Plasmid pET45b-*P. horikoshii* RadA (Δ1-115 codons) and pACYC-*P. horikoshii* MIG-RadAi were described previously (9, 13).

### Protein expression and purification.

*P. horikoshii* RadA, *P. horikoshii* RadA ΔNCR, and *T. sibericus* RadA were expressed from pET45b constructs in BL21(DE3) cells (Novagen). Cells were grown to mid-log phase (optical density at 600 nm of ~0.5) at 37°C and then switched to 16°C, and protein expression was induced by addition of 0.5 mM isopropyl β-d-1-thiogalactopyranoside (IPTG) for ~16 h. Proteins were purified by immobilized metal affinity chromatography as previously described (13). MIG-*P. horikoshii* RadAi, MIG-*P. horikoshii* RadAi ΔNCR, and MIG-*T*. *sibericus* RadAi were expressed from pACYC constructs in BL21(DE3) cells. Cells were grown to mid-log phase at 37°C, then switched to 30°C, and protein expression was induced for 1 h by addition of 0.5 mM IPTG. Cells were next pelleted by centrifugation at 4,000 × *g* for 10 min, lysed by sonication, and then 100 μg/ml spectinomycin was added to halt protein synthesis.

### Splicing assays.

Splicing assays were performed as previously described (13), with temperatures, times, and additions of ssDNA (M13mp18; New England Biolabs) as noted in the figures and their legends. Calculation of splicing in native exteins was done as previously described (13), except for comparison of *P. horikoshii* RadA to *P. horikoshii* RadA ΔNCR, for which the intein band was omitted from analysis due to lack of identification in the *P. horikoshii* RadA ΔNCR samples. For MIG-RadAi splicing assays, crude lysate was incubated for 1 h at 25°C following expression and lysis (above) and analyzed by in-gel fluorescence as described elsewhere (10). The extent of splicing was calculated as previously described (13). Samples in all cases were separated on 8-to-16% SDS-PAGE gels. In all splicing assays, densitometry using ImageJ (http://imagej.nih.gov/ij/) was used to calculate product levels. Data were plotted and curves were fit using Prism7 (GraphPad). Further information regarding our experimental procedures is provided in Text S1.

10.1128/mBio.02304-17.1 TEXT S1  Additional study results. Download TEXT S1, DOCX file, 0.03 MB. Copyright © 2018 Lennon et al. 2018 Lennon et al. This content is distributed under the terms of the Creative Commons Attribution 4.0 International license.

### Sequence alignment.

Clustal Omega was used to align the protein sequences of *P. horikoshii* and *T. sibericus* RadA proteins. Specifically, codons 116 to 529 of *P. horikoshii* RadA (O58001.1) and codons 116 to 526 of *T. sibericus* RadA (ACS91039.1) were aligned with sequences analyzed from UniProt.org.

### Structural modeling.

Ten *P. horikoshii* RadA wild-type precursor models reported in a previous study (9) were used as structural templates for generating 10 corresponding homology models for the *T. sibericus* RadA wild-type precursors. These precursor models contained 482 amino acid residues that spanned the range from Ser45 to Asp526 in wild-type *T. sibericus* RadA. The homology models were generated using the Modeller software (29) with the best model chosen using the DOPE (30) and GA341 (31) energy functions. Further minimization was performed identically for all 10 *T. sibericus* and *P. horikoshii* models by using the program CHARMM, version c35b3 (32, 33) with the CHARMM36 force field for proteins (34). All protein nonhydrogen atoms were harmonically restrained with a force constant of 1 kcal/mol/Å^2^, and the model was minimized using 5,000 steps-of-steepest-descent (SD) minimization with an energy change tolerance of 0.001 kcal/mol and SHAKE constraints (35) on all hydrogen atoms. Nonbonded interactions were calculated using 999.0-Å cutoff distances for van der Waals and electrostatic interactions, to ensure that all possible interactions were fully included. The restraint force constant was then reduced to 0.5 kcal/mol/Å^2^, and the same minimization was repeated. Finally, the restraint was removed altogether, and an unrestrained minimization was performed to obtain each final optimized precursor model. The interaction energy between residue backbone or side chain atoms was calculated by designating the atoms types C, N, O, CA, HA, and HN as backbone atoms and all other atoms as side chain atoms.
