# Supplementary figures and images for: Conditional Protein Splicing Switch in Hyperthermophiles through an Intein-Extein Partnership
Source: mBio. 2018 Jan 30;9(1):e02304-17. doi: 10.1128/mBio.02304-17 (PMC5790916; doi:10.1128/mBio.02304-17)

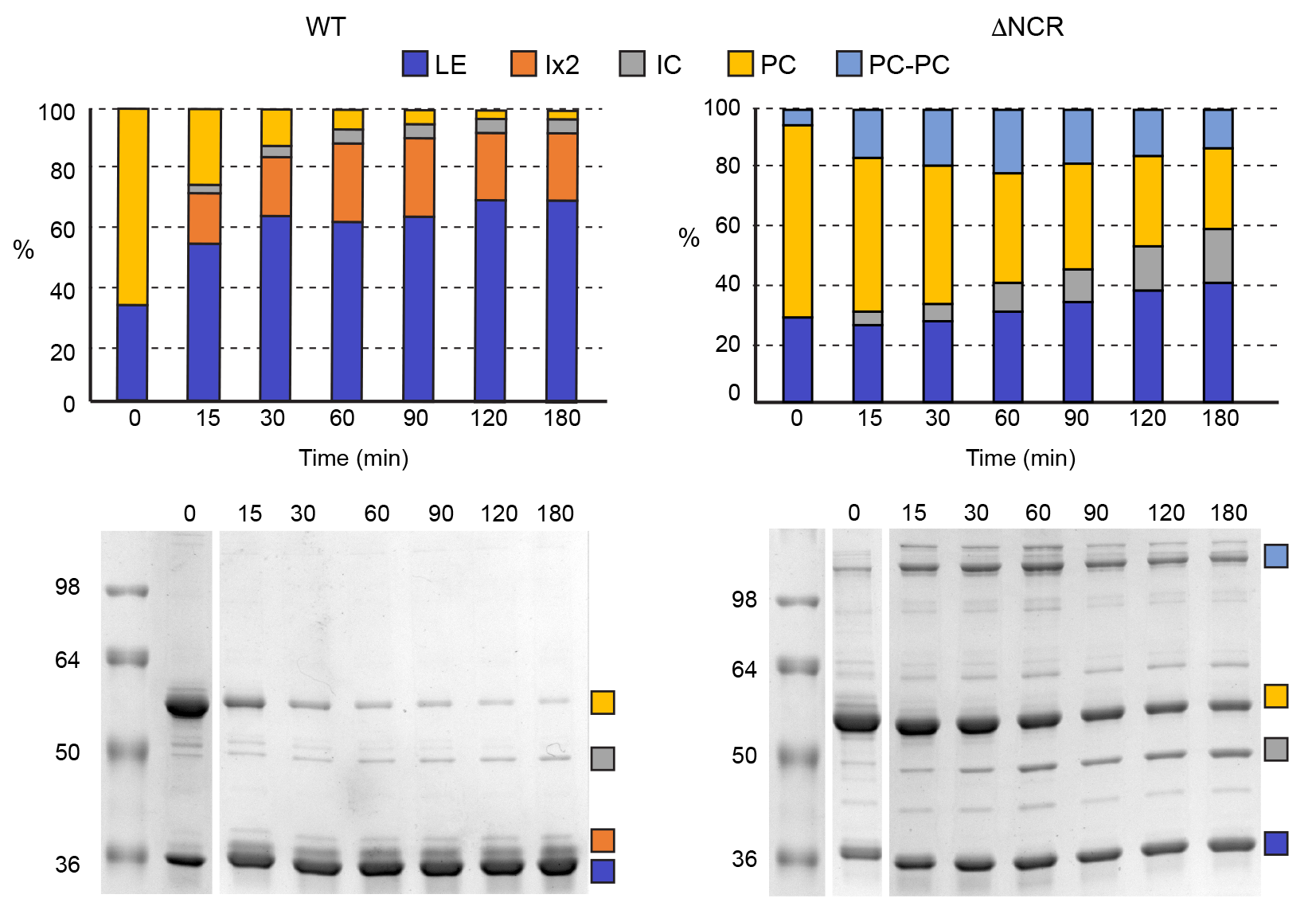

Supplement: FIG S1 [file mbo001183692sf1.tif]

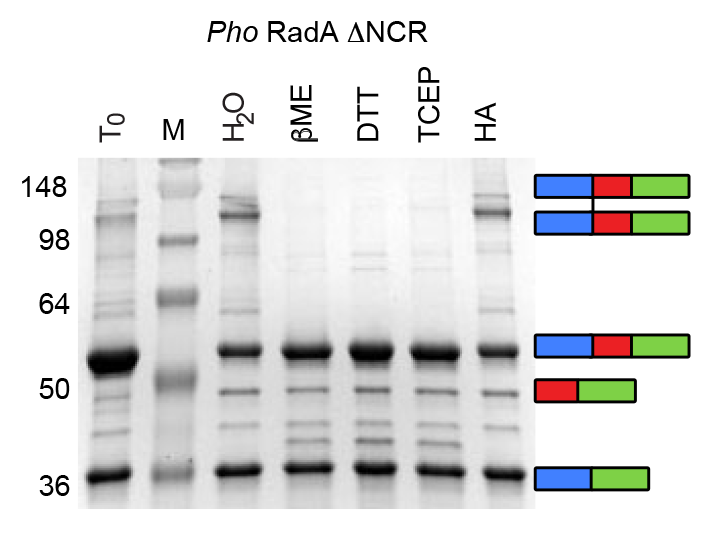

Supplement: FIG S2 [file mbo001183692sf2.tif]
