# Supplementary material for: Conditional Protein Splicing Switch in Hyperthermophiles through an Intein-Extein Partnership
Source: mBio. 2018 Jan 30;9(1):e02304-17. doi: 10.1128/mBio.02304-17 (PMC5790916; doi:10.1128/mBio.02304-17)
Supplement: TEXT S1 [file mbo001183692s1.docx]

**SUPPLEMENTAL MATERIALS**

**Conditional protein splicing switch in hyperthermophiles through intein-extein partnership**

Christopher W. Lennon, Matthew Stanger, Nilesh K. Banavali, Marlene Belfort

**SUPPLEMENTAL RESULTS**

*Pho RadA ΔNCR precursor processes largely to off-pathway products*

We monitored splicing over a three-hour time-course at 75°C, a condition where *Pho* RadA wild-type splicing is almost complete after 30 min. (Fig. 1) (1). Overall, we observe significantly slower splicing, manifest in both disappearance of precursor and appearance of ligated exteins for *Pho* RadA ΔNCR compared to wild-type (Fig. S1). Lower levels of ligated exteins were observed for *Pho* RadA ΔNCR compared to wild-type at the end of the time-course even after >80% of the starting precursor had processed, indicating *Pho* RadA ΔNCR splices much less accurately than wild-type. Consistent with decreased accuracy, we observe greater accumulation of two prominent off-pathway products for *Pho* RadA ΔNCR. The first, previously identified as intein-C-extein (I-C) (2), results from N-terminal cleavage. After 3 h, >4-fold more I-C accumulates in *Pho* RadA ΔNCR than in wild-type (Fig. S1).

The second prominent off-pathway product observed, which we do not detect in *Pho* RadA wild-type splicing reactions, runs between the 98 kDa and 148 kDa markers. Based on the size of this new high molecular weight product, we reasoned it could be a dimer of precursors, formed by a disulfide bond between the catalytic C1 residues of two precursor proteins, as C1 is the only cysteine present in *Pho* RadA. To test this hypothesis, we treated samples with the nucleophiles dithiothreitol (DTT), β-mercaptoethanol (BME), *tris*(2-carboxyethyl)phosphine (TCEP) or hydroxylamine (HA). If the product is disulfide bonded, DTT, BME and TCEP should reduce the bond, while HA should not because it is a non-reducing nucleophile (3). We observe that DTT, BME and TCEP treatment indeed lead to the disappearance of the high molecular weight band, along with an increase in the amount of PC, whereas water or HA had no effect (Fig. S2).

**SUPPLEMENTAL REFERENCES**

1. **Topilina NI, Novikova O, Stanger M, Banavali NK, Belfort M.** 2015. Post-translational environmental switch of RadA activity by extein-intein interactions in protein splicing. Nucleic Acids Res **43:**6631-6648.

1. **Lennon CW, Stanger M, Belfort M.** 2016. Protein splicing of a recombinase intein induced by ssDNA and DNA damage. Genes Dev **30:**2663-2668.
2. **Callahan BP, Topilina NI, Stanger MJ, Van Roey P, Belfort M.** 2011. Structure of catalytically competent intein caught in a redox trap with functional and evolutionary implications. Nature Struct. & Mol. Bio. **18:**630-633.

**SUPPLEMENTAL FIGURE LEGENDS**

**Supplemental Figure 1**: *Pho* RadA ΔNCR forms primarily off-pathway products. Reactions were performed at 75°C for the indicated times. Top: Stack plot displays the level of splicing precursor (PC) and products (PC-PC, precursor dimer; I-C, Intein-C-extein; Ix2, Intein dimer; LE, ligated exteins) as 100% over time. Bottom: Gel of stack plot above. Samples were separated, analyzed, and identified as in Fig. 1.

**Supplemental Figure 2**: *Pho* RadA ΔNCR forms an intermolecular precursor-precursor disulfide bond at intein C1 residues. Following incubation of *Pho* RadA ΔNCR for 25 min. at 75°C to generate the high molecular weight product, the reaction was divided and either DTT, BME, TCEP, HA or water was added. To allow for disulfide reduction, reactions were set at 25°C for 80 min. Reactions were separated on 8-16% SDS-PAGE gels and stained with Coomassie.
